# Association between witnessing physical violence between parents and intimate partner violence against Bolivian men: A national cross-sectional analysis of the 2016 demographic and health survey

**DOI:** 10.1016/j.pmedr.2024.102948

**Published:** 2024-12-16

**Authors:** J. Matias Bardales-Rodríguez, Flavia Rioja-Torres, Akram Hernández-Vásquez, Diego Azañedo

**Affiliations:** a CHANGE Research Working Group, Universidad Científica del Sur, Lima, Peru; b Centro de Excelencia en Investigaciones Económicas y Sociales en Salud, Vicerrectorado de Investigación, Universidad San Ignacio de Loyola, Lima, Peru; c Faculty of Health Sciences, Universidad Científica del Sur, Lima, Peru

**Keywords:** Gender-based violence, Men, Adverse childhood experiences, Surveys and questionnaires, Bolivia

## Abstract

**Objective:**

Assess the association between having witnessed physical violence between parents and intimate partner violence (IPV) against men in Bolivian adults according to the Encuesta de Demografia y Salud (EDSA) 2016.

**Methods:**

A retrospective cross-sectional study was conducted using data from the EDSA 2016 in Bolivia. The variable of interest in this study was IPV in men experienced during the last 12 months (any type of violence, physical and/or sexual, and psychological). The exposure variable was having witnessed physical violence between parents. Unadjusted and adjusted generalized linear models were constructed to assess the association of interest, and prevalence ratios (PR) with 95 % confidence intervals (95 %CI) were reported.

**Results:**

Witnessing physical aggression between parents in childhood was associated with a greater probability of suffering intimate partner violence in adulthood (adjusted PR [aPR]: 1.50; 95 %CI: 1.34–1.69). Similarly, the presence of physical aggression between parents in childhood was associated with a higher probability of physical and/or sexual violence (aPR: 1.92; 95 %CI: 1.53–2.39) and psychoverbal violence (PR: 1.48; 95 %CI: 1.32–1.67). The association identified was not modified by having suffered violence during childhood.

**Conclusions:**

Participants who witnessed physical violence between parents were more likely to suffer intimate partner violence (IPV), psycho-verbal violence and physical and/or sexual violence by their partners.

## Highlights

• Intimate partner violence is a public health problem that also affects men.
• Witnessing physical violence between parents has a negative impact on adulthood.
• Bolivian men are a great risk to suffer physical, psychoverbal, and sexual violence.

## 1 Introduction

The Center for Disease Control and Prevention (CDC) in the USA considers intimate partner violence (IPV) to be a serious health problem. The CDC defines IPV as abuse or aggression including physical violence, sexual violence, stalking or psychological violence that occurs in a romantic relationship (Centers for Disease Control and Prevention, 2021). According to figures from the 2022 CDC National Intimate Partner and Sexual survey, 41 % of women and 26 % of men experienced physical, sexual or stalking violence by an intimate partner, while 49.4 % and 45.1 % experienced psychological aggression (Leemis et al., 2022). Many studies also mention a high prevalence of physical violence against men with values from 3.4 % to 20.3 %, psychological violence from 7.3 % to 37 % and sexual violence from 0.2 to 7 % in North America, Europe, Africa and Asia (Kolbe and Büttner, 2020).

A systematic review in Latin America, including studies from Mexico, Peru, Brazil, and Ecuador published between 2000 and 2021, showed that violence against men among heterosexual couples has increased, with rates ranging from 13 % to 71 %. (Rojas-Solís et al., 2019). In Bolivia, 9 % of men reported suffering physical aggression by their partner. (Camargo, 2019). IPV has individual and social consequences, among which the most prevalent are death of the victims, addictions, associated health and judicial costs and loss of productivity at work. (Niolon et al., 2017). Thus, effective public health measures are required to address their occurrence and potential harms.

Many studies have identified associated factors related to IPV, such as disability, substance abuse, age, education, among others. (Breckenridge et al., 2019; Cowlishaw et al., 2022; Dardis et al., 2015; Dowling et al., 2016; Kamimura et al., 2016; Kim and Schmuhl, 2020; Lövestad and Krantz, 2012; Malik and Nadda, 2019; Mitra et al., 2016; Nowinski and Bowen, 2012). In particular, it has been found that among men, being older, married, and having a low level of literacy increase the probability of experiencing IPV (Spencer et al., 2022). Furthermore, people who experienced physical and sexual violence during childhood are more likely to accept any type of violence by their intimate partner in adulthood (Cowlishaw et al., 2022; Gubi and Wandera, 2022; Lövestad and Krantz, 2012; Mulawa et al., 2018; Nowinski and Bowen, 2012; Spencer et al., 2022). Also, in Latin American countries such as Peru, data indicate that men who suffered physical violence in childhood are more likely to be assaulted by their intimate partners (Fiestas et al., 2012).

Studies have identified the experience of violence in childhood as a factor associated with IPV in adulthood but have not assessed if witnessing physical violence between parents alone is also associated with being a victim of IPV in adulthood, and whether or not this association can be modified by having suffered parental violence directly. Exposure to this type of violent acts could generate a perception of normalization among the victims and increase the probability of suffering IPV in the future (Gubi and Wandera, 2022). However, some studies demonstrate that men experience and respond to childhood trauma, including violence, in ways that differ from women due to gender norms and expectations (Mbilinyi et al., 2012; Thompson et al., 2004). Also, men who experience childhood violence may face unique challenges, such as stigma or a lack of available resources tailored to their needs (Mbilinyi et al., 2012). In this sense, more research is needed to understand the relationship between exposure to parental violence and IPV in adulthood, and how the negative effects of this exposure on the mental and physical health of male victims can be prevented and treated.

This study aimed to evaluate the association between having witnessed physical violence between parents and partner violence against men in Bolivian adults according to data from the Encuesta de Demografia y Salud (EDSA) 2016.

## 2 Materials and methods

### 2.1 Context of study

Bolivia, a country located in South America, lies between the geographic coordinates of 57°26′ and 69°38′ west longitude of the Greenwich meridian and the parallels 9°38′ and 22°53′ south latitude. It borders with Brazil to the north and east, Argentina to the south, Peru to the west, Paraguay to the southeast, and Chile to the southwest (Instituto Nacional de Estadística, 2020). Its territorial surface covers 1,098,581 km^2^ (Instituto Nacional de Estadística, 2020).

In 2016, Bolivia had a population of 11,016,438 inhabitants, of which 5,536,112 were men (Instituto Nacional de Estadística, 2020b). The birth rate was 22 per 1000 inhabitants (World Bank, 2023a), while the infant mortality rate was 25 per 1000 children under five years old (World Bank, 2023b). In 2021, the life expectancy was 68 years (World Bank, 2023c).

### 2.2 Study design and population

The EDSA 2016 is a cross-sectional survey designed to provide information to evaluate policies in the health sector and establish a baseline for new strategies, plans, and programs. This survey constitutes the sixth edition of the series of Demographic and Health Surveys (DHS) carried out previously in 1989, 1994, 1998, 2003 and 2008. (International Household Survey Network, 2019).

The EDSA 2016 consists of three questionnaires: the Household Questionnaire, the Individual Questionnaire for Women, and the Individual Questionnaire for Men. Data collection was carried out between May and September 2016, through face-to-face interviews conducted by trained personnel accredited by National Institute of Statistics (INE) Bolivia (International Household Survey Network, 2019).

The EDSA 2016 targeted households in private dwellings across Bolivia's nine departments, including urban and rural areas (International Household Survey Network, 2019). Data were collected from women aged 14–49, children under five, and men aged 15–64. Collective housing residents, such as those in hospitals, hotels, prisons, military facilities, nursing homes, and schools, were excluded. The sampling frame was based on the 2012 Population and Housing Census, the 2010–2012 Multipurpose Cartographic Update, and the 2013 National Agricultural Census (International Household Survey Network, 2019).

The sampling frame covers the entire national territory and codes the geographic areas in primary sampling units, which are clearly defined in dispersed rural areas, clustered rural areas, and urban and peripheral areas (populated centers that are both in urban areas as rural). The secondary sampling units correspond to the dwellings, covering all private dwellings.

The EDSA uses a two-stage probability sampling stratified by conglomerates in the first stage. Selections in the first stage were made with probabilities proportional to the size of the number of dwellings, while in the second stage a fixed sample of dwellings was selected using a systematic approach. In addition, a household subsample was used to collect information on all eligible men in the selected or subsampled households.

The total sample size was 15,184 households, with the EDSA 2016 achieving response rates of 96.8 % in urban areas, 99.3 % in rural areas, and 97.8 % overall. For the weights or expansion factors, design weights were calculated based on the sampling probabilities for each stage and conglomerate. In the survey, the following weighting factors were applied: women factor, men factor and household factor. Further details can be found in the 2016 EDSA Sample Design Document and Final Report (Instituto Nacional de Estadística, 2017).

For the present study, the original sample consisted of 4975 men aged 15 years or older. Among them, 3029 were currently married or cohabiting with a woman, representing the target population. From this group, 42 individuals with missing data on the parental aggression variable were excluded. Additionally, 4 participants under the age of 18 were removed. Consequently, the final analytical sample consisted of 2983 individuals.

### 2.3 Variables

#### 2.3.1 Outcome

The variable of interest in this study was IPV experienced by men during the last 12 months, which was obtained from 13 questions (See Supplementary data).

If the respondent answered affirmatively to the options of “very often”, “sometimes”, “once”, we assume that the respondent had been a victim of IPV in the last 12 months. Then, we generated two dichotomous variables that identify the type of violence suffered by the man, i.e., physical and/or sexual, and psychological (0 = No, 1 = Yes).

#### 2.3.2 Exposure

The exposure variable was having witnessed physical violence between parents. This variable was created from the question Did you ever observe physical aggression between your parents? Responses were dichotomized into 0 = No and 1 = Yes.

#### 2.3.3 Potential confounders

Potential confounders included individual, socioeconomic, and contextual characteristics: age (18 to 35, 36 to 59, 60 to 64 years), educational level (up to primary, secondary, higher), marital status (married, cohabiting), disability (no, yes), indigenous (no, yes), worked all year (no, yes), sexually transmitted infections (no/does not know, yes), alcohol, having smoked in the last 12 months (no, yes), area of residence (urban, rural), and department of residence (Pando, Chuquisaca, La Paz, Cochabamba, Oruro, Potosí, Tarija, Santa Cruz, Beni).

### 2.4 Statistical analysis

The weights assigned to each sample and the sample design characteristics were used to obtain all estimates, while the unweighted sample size (n) is presented for descriptive purposes. Descriptive statistics and cross-tabulations were calculated for the variables investigated, presenting absolute frequencies and weighted proportions for qualitative variables. To assess the magnitude and direction of the relationship between having witnessed physical violence between parents and IPV against men and its types, unadjusted and adjusted analyses were performed using generalized linear models of the Poisson family and with logit link. These models were performed in order to obtain unadjusted (PR) and adjusted (aPR) prevalence ratios together with their corresponding 95 % confidence intervals (CI). The adjusted analysis included possible confounding factors following epidemiological criteria. For the identification of these variables, a directed acyclic graph (DAG) was designed in the Daggity web software (Textor et al. 2011). In addition, an evaluation of effect modification of the variable “having suffered physical violence in childhood” was proposed, based on the introduction of an interaction factor in the regression models adjusted for the outcomes of general IPV, physical and/or sexual violence, and psychoverbal violence. Subsequently, the presence of interaction was verified using the testparm postestimation command in Stata.

All the statistical analyses were performed with Stata 17.0 statistical software (StataCorp, College Station, TX, USA). A *p* value <0.05, considering a two-tailed test, was used to determine statistical significance.

## 3 Results

Of a total of 2983 participants included in the analysis, the highest proportions corresponded to the age group 36–59 years (55.5 %), secondary education level (40.9 %), married marital status (65 %), living in an urban area (64.5 %) and in the department of Santa Cruz (29.3 %). Likewise, the majority did not have disabilities (97.2 %), worked during the year prior to the survey (78.3 %), did not have sexually transmitted infections (98.7 %), drank alcohol (73.9 %) and did not smoke during the last year (63.3 %) (Table 1).

**Table 1:** Sociodemographic and behavioral characteristics of Bolivian men included in the study, based on the 2016 Demographic and Health Survey (*n* = 2983).

Characteristics	n	%⁎
Age group		
18–35 years	1154	38.2
36–59 years	1630	55.5
60–64 years	199	6.4

Education level		
Elementary	1078	32.3
High school	1231	40.9
College/University	674	26.8

Marital status		
Married	1932	65.0
Cohabiting	1051	35.0

Disability		
No	2896	97.2
Yes	87	2.8

Indigenous		
No	1592	54.4
Yes	1391	45.6

Worked all year		
No	704	21.7
Yes	2279	78.3

Sexually transmitted infections		
No/ Don't know	2949	98.7
Yes	34	1.3

Alcohol in the last 12 months		
No	755	26.1
Yes	2228	73.9

Smoked in the last 12 months		
No	1821	63.3
Yes	1162	36.7

Area of residence		
Urban	1691	64.5
Rural	1292	35.5

Residence department		
Pando	117	1.3
Chuquisaca	238	5.1
La Paz	563	25.1
Cochabamba	446	16.8
Oruro	248	5.1
Potosí	314	8.0
Tarija	237	4.9
Santa Cruz	561	29.3
Beni	259	4.4

n: unweighted sample size.

⁎ Percentages include the weights and EDSA sample specification.

The highest prevalence of IPV was identified in the 18–35 age group (44.3 %), in individuals with a higher education (42.1 %), cohabitants (41.1 %), without disability (34.6 %), non-indigenous (35.7 %), those without work in the last year (35. 6 %), those who had had a sexually transmitted infection (66.0 %), those who drank alcoholic beverages (37.7 %) and smoked (42.1 %) in the last 12 months, urban residents (40.1 %), and residents of Oruro (52.8 %) (Table 3). The frequencies of physical and/or sexual violence were higher in the same categories as those found for IPV, except for educational level, with which the highest frequency was identified at the secondary level (16.1 %), and the department of residence, with the highest proportion being identified in Pando (18.7 %). Similarly, the frequencies of psychoverbal violence were higher in the same categories as IPV, except that the highest frequency was found in the group with disabilities (Table 2).

**Table 2:** Characteristics of Bolivian men by type of intimate partner violence experienced, based on the 2016 Demographic and Health Survey.

	Intimate partner violence		Physical and/or sexual violence		Psychoverbal violence	
	No (*n* = 1962)	Yes (*n* = 1021)	No (*n* = 2600)	Yes (*n* = 383)	No (*n* = 2009)	Yes (*n* = 974)
Characteristics	% (95 % CI)	% (95 % CI)	% (95 % CI)	% (95 % CI)	% (95 % CI)	% (95 % CI)
Overall	65.4 (62.8–67.9)	34.6 (32.1–37.2)	86.6 (84.9–88.2)	13.4 (11.8–15.1)	66.9 (64.3–69.)	33.1 (30.6–35.7)
Age group						
18–35 years	55.7 (51.9–59.4)	44.3 (40.6–48.1)	83.4 (80.3–86.1)	16.6 (13.9–19.7)	57.2 (53.5–60.9)	42.8 (39.1–46.5)
36–59 years	69.9 (66.7–72.9)	30.1 (27.1–33.3)	87.9 (85.7–89.8)	12.1 (10.2–14.3)	71.4 (68.3–74.4)	28.6 (25.6–31.7)
60–64 years	84.2 (77.2–89.4)	15.8 (10.6–22.8)	94.6 (90.5–97.0)	5.4 (3.0–9.5)	84.9 (77.9–89.9)	15.1 (10.1–22.1)

Educational level						
Elementary	76.1 (72.3–79.5)	23.9 (20.5–27.7)	91.5 (89.2–93.4)	8.5 (6.6–10.8)	77.2 (73.4–80.6)	22.8 (19.4–26.6)
High school	61.9 (58.3–65.3)	38.1 (34.7–41.7)	83.9 (81.1–86.3)	16.1 (13.7–18.9)	63.6 (60.0–67.1)	36.4 (32.9–40.0)
College/University	57.9 (52.9–62.8)	42.1 (37.2–47.1)	84.9 (81.0–88.1)	15.1 (11.9–19.0)	59.4 (54.4–64.2)	40.6 (35.8–45.6)

Marital status						
Married	68.9 (65.9–71.7)	31.1 (28.3–34.1)	88 (86.0–89.7)	12 (10.3–14.0)	70.4 (67.4–73.2)	29.6 (26.8–32.6)
Cohabiting	58.9 (54.4–63.2)	41.1 (36.8–45.6)	84.1 (80.9–86.8)	15.9 (13.2–19.1)	60.3 (55.9–64.5)	39.7 (35.5–44.1)

Disability						
No	65.4 (62.7–67.9)	34.6 (32.1–37.3)	86.5 (84.8–88.1)	13.5 (11.9–15.2)	66.9 (64.2–69.4)	33.1 (30.6–35.8)
Yes	66.2 (53.7–76.8)	33.8 (23.2–46.3)	89.2 (80.1–94.4)	10.8 (5.6–19.9)	66.8 (54.2–77.3)	33.2 (22.7–45.8)

Indigenous						
No	64.3 (60.9–67.6)	35.7 (32.4–39.1)	86.5 (84.2–88.6)	13.5 (11.4–15.8)	65.7 (62.2–68.9)	34.3 (31.1–37.8)
Yes	66.6 (62.9–70.2)	33.4 (29.8–37.1)	86.7 (84.2–88.8)	13.3 (11.2–15.8)	68.3 (64.6–71.8)	31.7 (28.2–35.4)

Worked all year						
No	64.4 (59.2–69.2)	35.6 (30.8–40.8)	85.9 (82.3–88.9)	14.1 (11.1–17.7)	66.5 (61.5–71.1)	33.5 (28.9–38.5)
Yes	65.7 (62.8–68.4)	34.3 (31.6–37.2)	86.8 (84.8–88.6)	13.2 (11.4–15.2)	67.0 (64.1–69.7)	33.0 (30.3–35.9)

Sexually transmitted infections						
No/ Don't know	65.8 (63.2–68.3)	34.2 (31.7–36.8)	87 (85.3–88.5)	13 (11.5–14.7)	67.3 (64.7–69.8)	32.7 (30.2–35.3)
Yes	34.0 (18.3–54.3)	66.0 (45.7–81.7)	57.7 (37.7–75.4)	42.3 (24.6–62.3)	34.0 (18.3–54.3)	66.0 (45.7–81.7)

Alcohol in the last 12 months						
No	74.1 (70.0–77.9)	25.9 (22.1–30.0)	91.9 (89.4–93.8)	8.1 (6.2–10.6)	75.3 (71.2–79.0)	24.7 (21.0–28.8)
Yes	62.3 (59.3–65.3)	37.7 (34.7–40.7)	84.8 (82.6–86.7)	15.2 (13.3–17.4)	63.9 (60.8–66.8)	36.1 (33.2–39.2)

Smoked in the last 12 months						
No	69.7 (66.6–72.6)	30.3 (27.4–33.4)	89 (86.9–90.8)	11 (9.2–13.1)	71.1 (68.0–73.9)	28.9 (26.1–32.0)
Yes	57.9 (54.1–61.7)	42.1 (38.3–45.9)	82.5 (79.5–85.2)	17.5 (14.8–20.5)	59.6 (55.8–63.3)	40.4 (36.7–44.2)

Area of residence						
Urban	59.9 (56.4–63.2)	40.1 (36.8–43.6)	83.4 (81.0–85.6)	16.6 (14.4–19.0)	61.6 (58.1–64.9)	38.4 (35.1–41.9)
Rural	75.4 (71.8–78.7)	24.6 (21.3–28.2)	92.4 (90.4–94.1)	7.6 (5.9–9.6)	76.5 (73.0–79.7)	23.5 (20.3–27.0)

Residence department						
Pando	53.2 (43.1–63.1)	46.8 (36.9–56.9)	81.3 (70.1–89.0)	18.7 (11.0–29.9)	58.8 (49.5–67.5)	41.2 (32.5–50.5)
Chuquisaca	67.6 (58.9–75.2)	32.4 (24.8–41.1)	89.1 (83.0–93.2)	10.9 (6.8–17.0)	67.9 (59.3–75.5)	32.1 (24.5–40.7)
La Paz	69.9 (64.6–74.8)	30.1 (25.2–35.4)	84.2 (80.1–87.5)	15.8 (12.5–19.9)	72.8 (67.6–77.5)	27.2 (22.5–32.4)
Cochabamba	64.6 (58.4–70.4)	35.4 (29.6–41.6)	90.8 (86.9–93.5)	9.2 (6.5–13.1)	65.0 (58.7–70.8)	35.0 (29.2–41.3)
Oruro	47.2 (38.2–56.3)	52.8 (43.7–61.8)	88.9 (83.5–92.7)	11.1 (7.3–16.5)	47.5 (38.5–56.6)	52.5 (43.4–61.5)
Potosí	64.3 (56.3–71.7)	35.7 (28.3–43.7)	87.6 (82.7–91.2)	12.4 (8.8–17.3)	67.7 (59.7–74.7)	32.3 (25.3–40.3)
Tarija	70.6 (61.8–78.1)	29.4 (21.9–38.2)	89.4 (82.1–94.0)	10.6 (6.0–17.9)	71.7 (62.8–79.2)	28.3 (20.8–37.2)
Santa Cruz	63.0 (57.0–68.7)	37.0 (31.3–43.0)	84.9 (80.8–88.3)	15.1 (11.7–19.2)	63.6 (57.6–69.3)	36.4 (30.7–42.4)
Beni	76.2 (68.0–82.9)	23.8 (17.1–32.0)	87.2 (81.8–91.1)	12.8 (8.9–18.2)	78.2 (70.3–84.5)	21.8 (15.5–29.7)

n: unweighted sample size.

Estimates include the weights and EDSA sample specifications.

CI: confidence interval.

The highest frequencies of having witnessed physical aggression among parents were found in the age groups 36 to 59 years (42.8 %), having a secondary education level (44.1 %), being married (45.0 %), without disability (42.7 %), indigenous (50.8 %), individuals who did not work all year (45. 1 %), those who had a sexually transmitted infection (62.1 %), those who drank alcohol (43.8 %) and smoked (44.7 %) in the last 12 months, rural residents (45.2 %), and individuals who lived in the district of Cochabamba (55.0 %) (Table 3).

**Table 3:** Characteristics of Bolivian men by exposure to physical aggression between parents, based on the 2016 Demographic and Health Survey.

	Saw physical aggression between parents	
	No (*n* = 1748)	Yes (*n* = 1235)
Characteristics	% (95 % CI)	% (95 % CI)
Overall	57.4 (54.7–60.1)	42.6 (39.9–45.3)
Age group		
18–35 years	57.4 (53.3–61.3)	42.6 (38.7–46.7)
36–59 years	57.2 (54.0–60.3)	42.8 (39.7–46.0)
60–64 years	59.9 (51.2–68.1)	40.1 (31.9–48.8)

Educational level		
Elementary	57.5 (53.2–61.6)	42.5 (38.4–46.8)
HIgh school	55.9 (52.1–59.7)	44.1 (40.3–47.9)
College/University	59.7 (54.9–64.3)	40.3 (35.7–45.1)

Marital status		
Married	55.0 (52.1–58.0)	45.0 (42.0–47.9)
Cohabiting	61.9 (57.4–66.2)	38.1 (33.8–42.6)

Disability		
No	57.3 (54.5–60.0)	42.7 (40.0–45.5)
Yes	63.4 (49.8–75.1)	36.6 (24.9–50.2)

Indigenous		
No	64.4 (60.8–67.8)	35.6 (32.2–39.2)
Yes	49.2 (45.7–52.7)	50.8 (47.3–54.3)

Worked all the year		
No	54.9 (49.7–60.1)	45.1 (39.9–50.3)
Yes	58.1 (55.2–61.0)	41.9 (39.0–44.8)

Sexually transmitted infections		
No/Don't know	57.7 (54.9–60.4)	42.3 (39.6–45.1)
Yes	37.9 (21.1–58.2)	62.1 (41.8–78.9)

Alcohol in the last 12 months		
No	61.1 (56.1–65.8)	38.9 (34.2–43.9)
Sí	56.2 (53.3–59.0)	43.8 (41.0–46.7)

Smoked in the last 12 months		
No	58.7 (55.3–62.0)	41.3 (38.0–44.7)
Yes	55.3 (51.4–59.0)	44.7 (41.0–48.6)

Area of residence		
Urban	58.9 (55.2–62.5)	41.1 (37.5–44.8)
Rural	54.8 (50.9–58.7)	45.2 (41.3–49.1)

Residence department		
Pando	68.1 (54.9–78.9)	31.9 (21.1–45.1)
Chuquisaca	63.9 (55.1–71.9)	36.1 (28.1–44.9)
La Paz	48.8 (43.9–53.8)	51.2 (46.2–56.1)
Cochabamba	45.0 (39.4–50.6)	55.0 (49.4–60.6)
Oruro	52.0 (43.5–60.5)	48.0 (39.5–56.5)
Potosí	54.1 (46.7–61.3)	45.9 (38.7–53.3)
Tarija	74.2 (68.4–79.3)	25.8 (20.7–31.6)
Santa Cruz	68.1 (62.0–73.6)	31.9 (26.4–38.0)
Beni	66.6 (56.9–75.0)	33.4 (25.0–43.1)

n: unweighted sample size.

Estimates include the weights and EDSA sample specifications.

CI: confidence interval.

Witnessing physical aggression between parents in childhood was associated with a greater probability of suffering IPV in adulthood (aPR: 1.50; 95 %CI: 1.34–1.69). Similarly, the presence of physical aggression between parents in childhood was associated with a higher probability of physical and/or sexual violence (aPR: 1.92; 95 %CI: 1.53–2.39) and psychoverbal violence (PR: 1.48; 95 %CI: 1.32–1.67) (Table 4). No statistical interaction was identified between having witnessed physical aggression between parents in childhood and having suffered violence as a child when evaluating general IPV (*p* = 0.273), physical and/or sexual violence (*p* = 0.593), and psychoverbal violence as an outcome (*p* = 0.222).

**Table 4:** Association between witnessing parental physical aggression and intimate partner violence among men in Bolivia, based on the 2016 Demographic and Health Survey.

Characteristics	Unadjusted model		Adjusted model⁎	
	PR (95 % CI)		aPR (95 % CI)	
Total violence				
Physical violence between parents				
No	1.00		1.00	
Yes	1.50 (1.33–1.70)		1.50 (1.34–1.69)	

Physical and/or sexual violence				
Physical aggression between parents				
No	1.00		1.00	
Yes	1.94 (1.55–2.44)		1.92 (1.53–2.39)	

Psychoverbal violence				
Physical aggression between parents				
No	1.00		1.00	
Yes	1.48 (1.30–1.68)		1.48 (1.32–1.67)	

PR: prevalence ratio; aPR: adjusted prevalence ratio; CI: confidence interval.

Estimates include the weights and EDSA sample specifications.

⁎ Adjusted for age, educational level, marital status, disability, ethnicity, work, sexually transmitted infections, alcohol in the last twelve months, smoked in the last year, area of residence, and department of residence.

## 4 Discussion

The present study evaluated the association between having witnessed physical violence between parents and the probability of suffering partner violence towards men. According to the Social Learning Theory or the Cycle of Violence, children who witness or experience violence are more likely to replicate these behaviors in their own relationships as adults. These theories provide a structured lens through which early exposure to violence influences adult behavior, including the likelihood of becoming either a victim of IPV (Copp et al., 2019; Wright and Fagan, 2013).

The study showed that 42.6 % of the men surveyed reported having witnessed physical aggression between parents, while 34.6 % of the respondents reported having suffered violence by their partner. Likewise, it was determined that there is a greater probability of suffering IPV, as well as physical and/or sexual and psychoverbal violence, in men with a history of witnessing physical violence between their parents. The association identified was not modified by having suffered violence during childhood.

Four out of 10 study participants reported witnessing physical violence between their parents. This figure is higher than that reported by Burazeri et al. in 2009 in undergraduate medical students of the only public medical university in Albania, in which the proportion of male witnesses of physical violence from father to mother in childhood and adolescence was reported to be 28.5 % (Burazeri et al. 2011). However, the sample used in this study was significantly smaller compared to our study and is clearly not representative of the entire population of Albania, which, being a European country, has distinctive characteristics from those of Bolivia. Another study conducted in adults between the ages of 18 and 29 years who attended college in South Texas between 2016 and 2019 reported a frequency like our study with 40.6 % of men witnessing violence from mother to father and 42.8 % from father to mother (Cano-Gonzalez et al. 2022). Exposure to physical violence in any of its types can cause psychological damage to the individual, evidenced through low self-esteem, poor school and work performance, and the normalization of violent events in their environment (Forke et al. 2019; Roustit et al. 2009). To avoid such consequences, it is necessary to implement strategies at the state level to recognize children, adolescents, and adults with dysfunctional families.

In our study, the frequency of having experienced IPV was 34.6 %. In this regard, Mellar et al. reported a prevalence of 49.9 % of any type of IPV in male respondents in the New Zealand Family Violence Study 2019 (NZFVS) survey. The difference identified may be because the NZFVS included economic abuse and controlling partner behavior as part of the measure of violence (Mellar et al. 2023), both of which may be quite common. In contrast, Worman and colleagues reported a prevalence of 6.2 % of male IPV victims during the years 2006 and 2016 in a forensic clinical examination center in Germany. However, the percentage reported in the previous study might be low compared to that of our study because male victims often do not report cases of IPV (Wörmann et al. 2021), leading to possible underreporting. Violence against men is not an uncommon problem and should be addressed to avoid its negative consequences at the individual and societal level. These consequences may include poor job performance, social isolation, and low personal fulfillment (Moore 2021). It is therefore necessary to identify, report and protect male victims of IPV, which is currently a challenging task due to the difficulties men may face in accepting and reporting having been victims.

Witnessing physical aggression between parents during childhood was found to be associated with an increased likelihood of IPV in general. This finding is like described in Uganda, where having witnessed father-to-mother violence increases the odds of suffering any type of IPV (OR = 1.42; 95 %CI: 1.14–1.76) (Gubi and Wandera, 2022). This association could be explained by the social learning theory, which postulates that experiencing violence throughout life can generate normalization of violent behaviors in adulthood (Smith-Marek et al. 2015). Likewise, our findings support the fact that violence can be transmitted from generation to generation by adopting normalization measures in men (Black et al. 2010); therefore, it is of utmost importance to be able to recognize households with family violence from the first level of care to avoid an increase of IPV cases.

The presence of physical aggression between parents was associated with a higher probability of physical and/or sexual violence by their partner. It is of note that IPV is more prevalent in low- compared to middle-income countries (Sardinha and Nájera Catalán 2018). This is because male victims may justify violent attitudes in their homes as demonstrated in a study conducted in 49 countries in which the proportion of men justifying domestic violence in Southeast and South Asia was 40.57 % compared to 32.74 % in countries located in the region of West and Central Asia and Europe. On the other hand, the frequency of suffering physical and/or sexual violence by a partner has been reported as being considerable in men with a history of violence in the family (50 %), low income (19.2 %) and dysfunctional family (15.4 %) (Gonzales Ruiz et al. 2023). The above is related to a greater tolerance of violence due to a lack of education about this problem and/or feeling inferior to a partner because of lower purchasing power.

Men who witnessed physical aggression between parents were more likely to experience psychoverbal violence by their partner. Similar findings were identified in a study of Ugandan men, in whom witnessing physical aggression from father to mother was associated with a higher odd of emotional violence (PR = 1.35; 95 %CI: 1.09–1.69) (Gubi and Wandera, 2022). This association is related to the theory that men often assume their gender as “the one who bears all and does not complain” learned from their father figures; therefore, they may perceive that, in the face of any verbal aggression from their partner, he would socially be seen as a “weak” man. Psychological mistreatment of men is one of the most frequent types of violence, due to unequal conditions such as having a better job or a lower level of education and the manipulation of the partner through blackmail or threats. For example, many men are ridiculed and belittled by their partner for not being “masculine” enough, for not earning enough money, or for being weak (Nybergh et al. 2016). If these acts are not adequately warned about and managed, they can generate negative health effects.

This study found that the association between witnessing parental physical aggression and IPV towards adult men was not modified by a history of childhood violence. This means that the association behaves in the same way in those who did or did not suffer violence in childhood. In this regard, we were not able to identify contrastable studies that have evaluated the same research question. Taking into consideration our results, in practical terms, violence prevention interventions should not only address the problem by preventing the occurrence of physical violence towards the child, but also by preventing the child from witnessing physical violence between parents. However, more longitudinal studies are needed to better understand the cause-and-effect relationship in this association.

This study has limitations, which deserve to be mentioned for adequate interpretation of its results. First, recall bias regarding parental or intimate partner violence could affect the accuracy of exposure and outcome frequencies. The cross-sectional design prevents determining causal relationships between exposure and outcomes. On the other hand, potential confounders included in the regression model may have changed significantly from childhood to adulthood, but these changes could not be accounted for due to the study's design. Other variables, such as duration of the relationship, parental education level and social support of the victims, could not be assessed due to their absence in the EDSA. Social desirability bias may also influence responses, as participants might underreport or misrepresent sensitive information. Despite these limitations, the use of EDSA 2016 ensures nationally representative estimates and provides valuable insights into IPV among adult men and its potential association with witnessing parental violence during childhood, a topic that remains understudied.

## 5 Conclusions

This study found that men who witnessed parental physical violence were more likely to experience physical, psychoverbal, and sexual violence from their partners. This exposure has negative repercussions on the life of men who are socially seen as a being without weakness or vulnerability, especially in developing countries. Thus, multisectoral policies are required to address this problem. Strategies to reduce IPV should adopt a global perspective rather than a gendered focus, ensuring adequate and safe support for male victims while considering social stereotypes.

## Funding

This research did not receive any specific grant from funding agencies in the public, commercial, or not-for-profit sectors.

## Ethical approval

Ethical approval and consent to participate and publish are not applicable to this study. The EDSA data are anonymized and are not considered confidential; therefore, no ethics committee approval was required to conduct the present study.

## CRediT authorship contribution statement

**J. Matias Bardales-Rodríguez:** Writing – review & editing, Writing – original draft, Investigation, Conceptualization. **Flavia Rioja-Torres:** Writing – review & editing, Writing – original draft, Investigation, Conceptualization. **Akram Hernández-Vásquez:** Writing – review & editing, Writing – original draft, Software, Methodology, Investigation, Formal analysis, Data curation, Conceptualization. **Diego Azañedo:** Writing – review & editing, Writing – original draft, Methodology, Investigation, Conceptualization.

## Declaration of competing interest

The authors declare that they have no known competing financial interests or personal relationships that could have appeared to influence the work reported in this paper.

## Data Availability

The data used in this study are publicly available from the National Institute of Statistics (INE) website (https://www.ine.gob.bo/).

## Appendix A Supplementary data

Supplementary material Image 1
