# Supplementary material for: Association between witnessing physical violence between parents and intimate partner violence against Bolivian men: A national cross-sectional analysis of the 2016 demographic and health survey
Source: Prev Med Rep. 2024 Dec 16;49:102948. doi: 10.1016/j.pmedr.2024.102948 (PMC11729684; doi:10.1016/j.pmedr.2024.102948)
Supplement: Supplementary file 1 — Supplementary material [file mmc1.docx]

**Supplementary material**

Variables used for type of violence by women towards men in the previous 12 months.

| **Question** | **Categories** | **Recoding** | **Type of violence** |
| --- | --- | --- | --- |
| Does she accuse you of being unfaithful? | Very often, sometimes, once or never. | 0=No, 1=Yes | Psychoverbal |
| Is she jealous of a friend? | Very often, sometimes, once or never. | 0=No, 1=Yes | Psychoverbal |
| Does she try to limit your contact with your family? | Very often, sometimes, once or never. | 0=No, 1=Yes | Psychoverbal |
| Does she humiliate or insult you? | Very often, sometimes, once or never. | 0=No, 1=Sí | Psychoverbal |
| Does she threaten to leave you? | Very often, sometimes, once or never. | 0=No, 1=Yes | Psychoverbal |
| Does she threaten to take your children away from you? | Very often, sometimes, once or never. | 0=No, 1=Yes | Psychoverbal |
| When angry, does she break objects in the home as a form of threat? | Very often, sometimes, once or never. | 0=No, 1=Yes | Physical and/or sexual |
| Does she hit your with her hand or kick you? | Very often, sometimes, once or never. | 0=No, 1=Yes | Physical and/or sexual |
| Have she pushed or pulled you? | Very often, sometimes, once or never. | 0=No, 1=Yes | Physical and/or sexual |
| Does she hit you with an object? | Very often, sometimes, once or never. | 0=No, 1=Yes | Physical and/or sexual |
| Has she tried to strangle or burn you? | Very often, sometimes, once or never. | 0=No, 1=Yes | Physical and/or sexual |
| Have you been forced to have sex that you did not want? | Very often, sometimes, once or never. | 0=No, 1=Yes | Physical and/or sexual |
| Have you been forced to engage in any type of sexual activity that you did not want? | Very often, sometimes, once or never. | 0=No, 1=Yes | Physical and/or sexual |
